# The interplay between soil structure, roots, and microbiota as a determinant of plant–soil feedback

**DOI:** 10.1002/ece3.2456

**Published:** 2016-10-05

**Authors:** Joana Bergmann, Erik Verbruggen, Johannes Heinze, Dan Xiang, Baodong Chen, Jasmin Joshi, Matthias C. Rillig

**Affiliations:** ^1^ Dahlem Centre of Plant Science (DCPS) Institute for Biology Freie Universität Berlin Berlin Germany; ^2^ Berlin‐Brandenburg Institute of Advanced Biodiversity Research (BBIB) Berlin Germany; ^3^ Department of Plant and Vegetation Ecology University of Antwerp Antwerp Belgium; ^4^ Department of Biodiversity Research Institute for Biochemistry and Biology University of Potsdam Potsdam Germany; ^5^ State Key Laboratory of Urban and Regional Ecology Research Center for Eco‐Environmental Sciences Chinese Academy of Sciences Beijing China; ^6^ College of Resources and Environment Qingdao Agricultural University Qingdao China

**Keywords:** arbuscular mycorrhizal fungi, biomass allocation, plant functional traits, plant–soil (belowground) interactions, soil aggregation, specific root length, succession, water‐stable aggregates

## Abstract

Plant–soil feedback (PSF) can influence plant community structure via changes in the soil microbiome. However, how these feedbacks depend on the soil environment remains poorly understood. We hypothesized that disintegrating a naturally aggregated soil may influence the outcome of PSF by affecting microbial communities. Furthermore, we expected plants to differentially interact with soil structure and the microbial communities due to varying root morphology. We carried out a feedback experiment with nine plant species (five forbs and four grasses) where the “training phase” consisted of aggregated versus disintegrated soil. In the feedback phase, a uniform soil was inoculated in a fully factorial design with soil washings from conspecific‐ versus heterospecific‐trained soil that had been either disintegrated or aggregated. This way, the effects of prior soil structure on plant performance in terms of biomass production and allocation were examined. In the training phase, soil structure did not affect plant biomass. But on disintegrated soil, plants with lower specific root length (SRL) allocated more biomass aboveground. PSF in the feedback phase was negative overall. With training on disintegrated soil, conspecific feedback was positively correlated with SRL and significantly differed between grasses and forbs. Plants with higher SRL were likely able to easily explore the disintegrated soil with smaller pores, while plants with lower SRL invested in belowground biomass for soil exploration and seemed to be more susceptible to fungal pathogens. This suggests that plants with low SRL could be more limited by PSF on disintegrated soils of early successional stages. This study is the first to examine the influence of soil structure on PSF. Our results suggest that soil structure determines the outcome of PSF mediated by SRL. We recommend to further explore the effects of soil structure and propose to include root performance when working with PSF.

## 1 Introduction

It has long been known that environmental context can have a profound influence on outcomes of biotic interactions, which can range from negative to positive. For instance, increasing facilitation among plants is found to correlate with environmental stress (He, Bertness, & Altieri, 2013), and soil fertility can change plant–mycorrhiza interactions from positive to negative (Johnson, 2010). One potentially important context for plant–soil biotic interactions is soil structure, as it can vary greatly depending on land‐use history, plant species composition, and successional stage (Barto, Alt, Oelmann, Wilcke, & Rillig, 2010; Erktan, Cécillon, Graf, & Roumet, 2015; Jastrow, Miller, & Lussenhop, 1998; Pérès et al., 2013). Soil structure is often described by aggregate measurements (e.g., water‐stable aggregates = WSA or mean weight diameter = MWD) as surrogates for the soil matrix. These aggregates have profound influences on nutrient cycling and soil organic matter dynamics through different physical processes (Six, Bossuyt, Degryze, & Denef, 2004). Furthermore, they determine the spatial structure of the environment in which soil microbial communities interact with plants. Studies examining the interaction between soil biota, plants, and aggregation mostly focus on unidirectional effects of the biota on the formation or destabilization of aggregates (Rillig & Mummey, 2006) rather than investigating the role of soil structure as a microhabitat. The function of soil structure possibly mediating the interaction between soil biota and plants as plant–soil feedback (PSF) has to our knowledge never been examined.

Conspecific or direct PSF is defined as the fitness effect a plant achieves from soil being modified in biotic and abiotic character by a plant of the same species (Bever, Westover, Antonovics, & Westover, 1997; van der Putten et al., 2013). Interactions between plants and soil biota can drive negative conspecific feedback (Bever et al., 1997; Kulmatiski, Beard, Stevens, & Cobbold, 2008) as in Janzen–Connell effects (Connell, 1971; Janzen, 1970), which can be strong enough to maintain plant coexistence (Bauer, Mack, & Bever, 2015; Bever, 2003; Heinze, Bergmann, Rillig, & Joshi, 2015; Mack & Bever, 2014; Mangan et al., 2010; van der Putten et al., 2013). In a meta‐analysis, it was found that feedbacks were generally stronger in artificial soil compared with field soil (Kulmatiski et al., 2008), which suggests the soil environmental context may affect feedbacks. Soil structure may be an important candidate: although a direct role of soil structure on PSF has been considered unlikely because effects of structure on plant growth will not be species specific (Ehrenfeld, Ravit, & Elgersma, 2005), it may strongly influence the soil biota responsible for the feedback effects. This can happen in multiple ways and will depend on whether there is a relationship between plant species specificity of soil biota on the one hand and their relative performance under different soil aggregation levels on the other. For fungal pathogens, we know that host specificity and virulence are negatively related to typical soil‐related lifestyles such as saprotrophic activities (Aguilar‐Trigueros, Powell, Anderson, Antonovics, & Rillig, 2014), and thus, the latter are expected to perform relatively better on more complex, aggregated soils as occurring under natural conditions. In cases of nutritionally based mutualists like arbuscular mycorrhizal fungi (AMF), we expect the opposite: because nutrients are sequestered inside soil aggregates reducing plant availability (Angers & Caron, 1998), interactions may become more positive under high soil aggregation levels. Both of these mechanisms would predict a less negative/more positive conspecific feedback under aggregated soils.

Apart from these soil biotic responses to soil structure and their projected effects on plants, plants may differ in their direct response to soil aggregation, which may in turn affect feedbacks. It has been argued that plant functional traits may have profound influences on PSF which has not been studied in detail yet (Baxendale, Orwin, Poly, Pommier, & Bardgett, 2014; van der Putten et al., 2013). Different plant functional types (PFT) such as grasses and forbs have been found to differ in feedbacks (Heinze et al., 2015; Kulmatiski et al., 2008), and it has been proposed that high specific root length (SRL) in grasses may increase susceptibility to pathogens (Bever, 1994; Newsham, Fitter, & Watkinson, 1995), while many forbs have higher reliance on mycorrhizal fungi for nutrient foraging (Reinhart, Wilson, Rinella, & Suding, 2012). Because PSFs are conceptually linked to a plant species‐specific modulation of soil (biota) by plant tissue, replacing high‐SRL roots by AM fungi as absorptive structures can be expected to elicit a reduction in negative feedback strength. Furthermore, it is known that the net effect of AMF on plant performance is highly dependent on environmental factors as well as the plants ability to acquire nutrients from soil (Johnson, Graham, & Smith, 1997). These different nutrient acquisition strategies may therefore determine direct plant responses to soil aggregation (e.g., allocation toward AMF versus fine roots).

**Figure 1:**
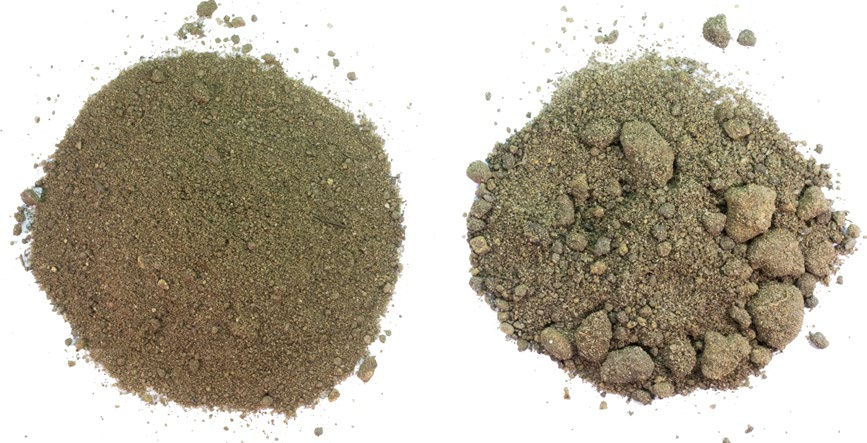
Disintegrated (left, MWD = 51 μm, 29% WSA) and aggregated soil (right, MWD = 109 μm, 44% WSA) used in the training phase

**Figure 2:**
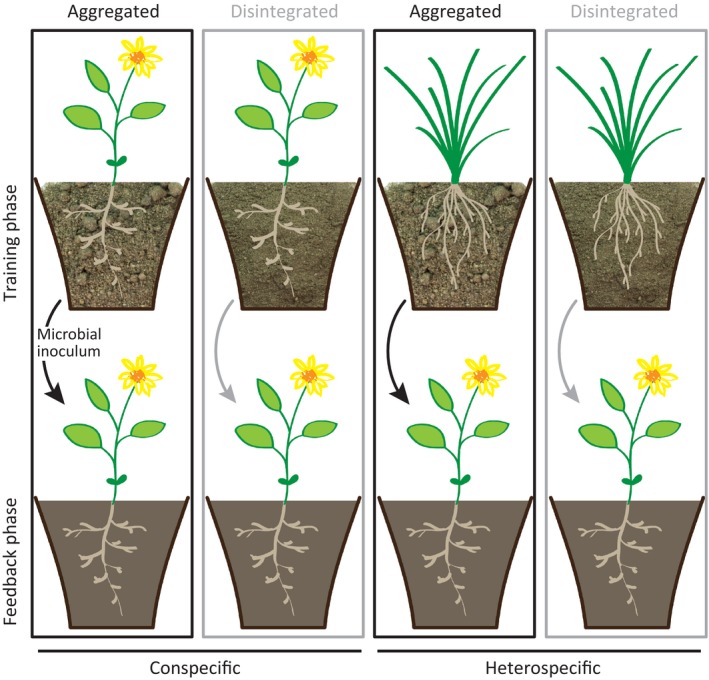
Experimental design. In the training phase, 10 plant species were grown on aggregated and disintegrated soil with eight replicates each. The microbial community of that trained soil was added to a common soil for the feedback phase. Nine species received conspecific inocula as well as nine different heterospecific inocula from both soil structure levels. This resulted in nine species × 2 soil histories (home/away) × 2 former soil structure (aggregated/disintegrated) × 9 replicates = 324 experimental units. Black and gray boxes represent the two different soil treatments “aggregated” and “disintegrated”

To investigate the effect of soil structure on feedback, we carried out a greenhouse feedback experiment including species‐specific root length as an explanatory variable. We tried to disentangle the physical and chemical effects of soil aggregation from the accompanying biological ones by first training the microbial community on different soil structure (Fig. 1) and then examining the effects of the resulting soil communities in a feedback phase on homogeneous soil (Fig. 2).

## 2 Materials and Methods

We conducted a classical feedback experiment where the training phase is meant to accumulate species‐specific soil biota which—in the feedback phase—affects different plant species.

### 2.1 Seed material

The seeds used for this experiment had been collected in the National Park Hainich (Central Germany) on different experimental grassland plots of the Biodiversity Exploratories (Fischer et al., 2010) in 2011. Grasses were *Anthoxantum odoratum* L.*, Briza media* L.*, Holcus lanatus* L., and *Dactylis glomerata* L., and forbs were *Plantago major* L.*, Plantago lanceolata* L.*, Centaurea jacea* L.*, Daucus carota* L.*, Leucanthemum vulgare* lam., and *Taraxacum officinale* F.H. wigg. All plants were perennials except for *D. carota*, which is a biennial (Kühn, Durka, & Klotz, 2004).

Seeds were surface‐sterilized in 7% sodium hypochlorite solution for 3 min (Bartelt‐Ryser, Joshi, Schmid, Brandl, & Balser, 2005) to avoid the input of species‐specific microbes.

### 2.2 Soil

We used fresh field soil from a loamy, sandy, mineral soil (Albic Luvisol; *N* = 0.12%, *C* = 1.87%, *C*/*N* ratio 15.58) from a meadow in Dahlem (Berlin, Germany), which has previously been used in experiments in our laboratory (Rillig et al., 2010; Salem, Kohler, Wurst, & Rillig, 2013). All species we used in the experiment (except *Briza media*) are common in the greater area. The soil was collected from approximately 10–30 cm depth, air‐dried, and then mixed and sieved (1 cm mesh). Then, half of it was disintegrated in a cement mill containing boulders (10 min per 15 l batch) to obtain the low‐aggregation‐status. The disintegrated soil had the same pH and only slightly elevated plant‐available phosphorus and nitrogen contents compared to the aggregated soil (see Table S1). We measured water‐stable aggregates (WSA) before (three replicates per soil structure level) and after (all replicates) the experiment by wet sieving using the method of Kemper and Rosenau (1986): 4.0 g of soil was weighed into sieves, rewetted through capillary action with deionized water, and sieved on a wet‐sieving apparatus (Eijkelkamp, Giesbeek, the Netherlands) for 3 min. The material left on the sieve was separated from coarse material (stones, organic debris), and both were weighed after drying at 60°C. MWD was measured to further characterize the structure of the soil prior to training according to Lehmann and Rillig (2013) by dry sieving of about 200 g soil (three replicates per soil structure level) through a stack of sieves (2 mm, 1 mm, 250 μm, 53 μm) and calculating the sum of the proportions of the weight and mean diameter of aggregates of the resulting five size classes.

### 2.3 Specific root length

To obtain the SRL as a species‐specific trait for our plant species not confounded by our soil treatment, we measured it on plants of an independent, earlier experiment (Heinze et al., 2015) that used the same seed material. They were grown in cones (0.41 L; Stueve & Sons; USA) on an autoclaved soil–sand mixture (40:60) with field soil from one less intensively managed pasture in the National Park Hainich from which most seeds originated. Plants were grown for 18 weeks in 2012 under greenhouse conditions and were then harvested and air‐dried for storage. In 2013, roots of three replicates per species were gently washed by hand, dried at 40°C for at least 3 days, and weighed to obtain dry weight. SRL was calculated using the WinRhizo scanner‐based system (Regents Instruments, Inc., Canada) (see mean values in Table S5).

### 2.4 Training phase

In a first experiment, set up from end of May until mid‐August 2013 for a duration of 10 weeks, we grew 10 plant species on aggregated (H: 44% WSA, MWD = 109 μm) versus disintegrated (L: 29% WSA, MWD = 51 μm) soil: the “training phase.” Seeds germinated on petri dishes on filter paper soaked in deionized water. Within a week after germination, seedlings were transplanted into rose pots containing 2.5 kg soil and set up in a randomized block design in a greenhouse (light: 16 hr, dark: 8 hr), watered daily for the first 4 weeks (50 ml tap water per day) using an automatic watering system after which they were watered manually additionally every second‐third day according to plant needs. The experimental setup consisted of two treatments (aggregated/disintegrated) * 10 species * 8 replicates resulting in 160 pots in total. The pots planted with *P. major* and *L. vulgare* contained two plant individuals instead of one because seedlings were frail at time of transplant but grew too vigorously to be thinned afterward. After harvest, we measured aboveground and belowground dry biomass as well as soil aggregation. Roots were taken out of the soil and gently washed by hand. To confirm that microbiota had not been destroyed during the disintegrating process, we measured arbuscular mycorrhizal (AM fungal) colonization in the roots using six replicates per species and treatment. We used ink and vinegar staining (Vierheilig & Coughlan, 1998) and counted 100 intersects per replicate under a light microscope to determine colonization rate by AM hyphae as well as arbuscules and vesicles (according to McGonigle, Miller, Evans, Fairchild, & Swan, 1990).

### 2.5 Feedback phase

The trained soil was air‐dried in closed paper bags and stored dry for <2 months at room temperature. This soil was used to produce the inoculum for the second phase of the experiment: the “feedback phase.” The inoculum was extracted by mixing 2.5 L soil (pooled from eight replicates for each species and soil structure level), with 5 L deionized water, stirring vigorously for 2 min, leaving it to settle for 1.5 min and decanting through a 212‐μm sieve. Inoculum was stored at 4°C and used within 48 hr. Nine replicates of each species received an inoculum trained by the same species (home or conspecific) and another nine received an inoculum each trained by one of the nine other species (away or heterospecific), which were all either from aggregated or disintegrated soil. *T. officinale* was used as donor plant for inoculum but not as receiver plant in the feedback phase in order to have the same number of replicates for home and away treatments. We used this well‐established experimental approach (Klironomos, 2002) to create a balanced design but are aware of the fact that each away species may have a different effect on the microbial community which can cause a higher variation in the away treatment (Van de Voorde, van der Putten, & Martijn Bezemer, 2011). This resulted in nine species (four grasses and five forbs) × 2 soil histories (home and away) × 2 soil structure levels (aggregated and disintegrated) × 9 replicates = 324 experimental units in a fully randomized design (for the experimental design see Fig. 2).

During the experiment, set up in October 2013, we used an autoclaved soil (aggregated field soil)‐sand mixture (50:50) and added the inoculum from the training phase. We did so to disentangle the biotic effects caused by the soil structure of the training phase from physical and chemical effects, which would have occurred using the whole trained soil. Seeds were surface‐sterilized and immediately sown on the soil–sand mixture covered with a thin layer of sterile sand. They were watered daily and transplanted into cones in the cotyledon stage while adding 10 ml of the inoculum which saturated the whole root area of the seedling. Plants were grown for 6 weeks to minimize the further effect on physical soil structure and to prevent the roots from getting pot bound and then harvested to measure aboveground and belowground dry biomass.

### 2.6 Statistical analysis

All statistical analyses were conducted using R version 3.0.3 (R Core Team 2014). In the training phase, we tested for effects of soil structure and SRL on (1) plant biomass (total biomass as well as aboveground biomass and belowground biomass separately), (2) biomass allocation (log(aboveground biomass/belowground biomass)) and (3) the percentage of water‐stable aggregates (WSA) after the training phase. Therefore, we fitted a general linear mixed‐effects model with effect‐coded variables using the package lmerTest (Kuznetsova, Brockhoff, & Christensen, 2014) and performed a type 3 ANOVA. The model contained the fixed effects “soil structure” (aggregated/disintegrated) and “SRL” (numerical) as well as the random effect “plant species.” For the analysis of the hyphal colonization, we fitted a general linear mixed‐effects model with the fixed effects “soil structure” and “SRL” and the random effects “plant species” and “person” (two persons were counting) and performed a type 3 ANOVA. Pearson's correlation coefficient was calculated to determine the relationship between the formation of WSA and the SRL.

In the feedback phase, we wanted to test for the effects of soil biota originating from different soil structure and trained by different plant species with varying SRL on (1) plant biomass (total biomass as well as aboveground biomass and belowground biomass separately) and (2) biomass allocation (log(aboveground biomass/belowground biomass)). To determine the feedback effect of “home versus away,” we fitted a general linear mixed‐effects model with the fixed effects “history (home/away)” and “former soil structure” (aggregated/disintegrated) and the random effect “species” (in the feedback phase) and performing a type 3 ANOVA (adapted from Brinkman, Van der Putten, Bakker, & Verhoeven, 2010). The “former soil structure” describes the initial soil conditions under which the microbial community was trained. To have a closer look into the effect of soil structure under different soil histories, we then split our dataset by soil history (home/away) and fitted a general linear mixed‐effects model performing a type 3 ANOVA, with the fixed effects “former soil structure” (aggregated/disintegrated) and “SRL” (numerical) and the random effect “plant species.” Pearson's correlation coefficient was calculated to determine the relationship between SRL and dry biomass production as well as biomass allocation.

As the trait “SRL” was collinear with the factor “PFT” (grasses/forbs), we could not analyze them simultaneously. The PFT is nevertheless an important predictor for plant performance. We therefore fitted all models containing the fixed effect (SRL) additionally with the factor “PFT” replacing the former one.

## 3 Results

### 3.1 Training phase

**Figure 3:**
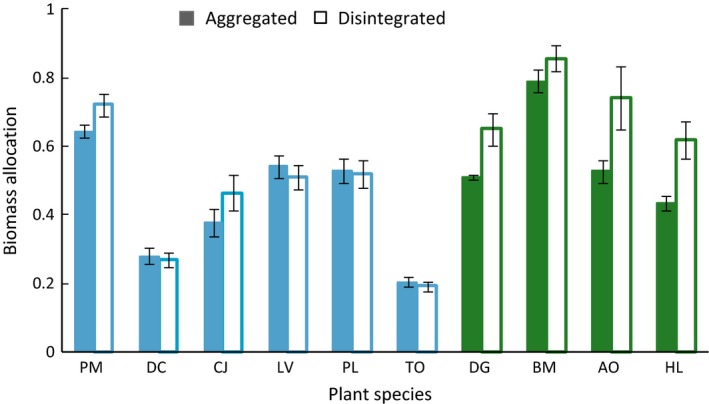
Training phase. Biomass allocation [log(aboveground biomass/belowground biomass)] on aggregated versus disintegrated soil. Species are sorted by specific root length (SRL) from low to high. Blue color indicates forbs and green color indicates grasses. Data represent mean ± SE. (PM) *Plantago major*, (DC) *Daucus carota*, (CJ) *Centaurea jacea*, (LV) *Leucanthemum vulgare*, (PL) *Plantago lanceolata*, (TO) *Taraxacum officinale,* (DG) *Dactylis glomerata*, (BM) *Briza media*, (AO) *Anthoxantum odoratum*, (HL) *Holcus lanatus*. The interaction between the effects “soil structure” and “SRL” significantly affects the biomass allocation with *p *= .004 in a linear mixed‐effects model with the random effect “plant species” (see Table 1)

**Figure 4:**
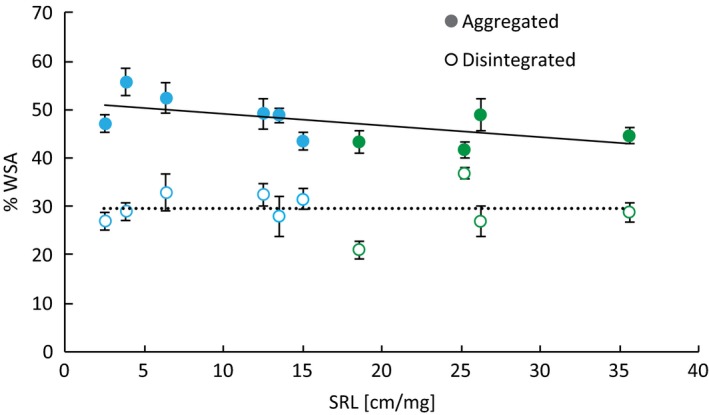
Training phase. Formation of water‐stable aggregates (WSA) on the two soil structure levels in correlation with the specific root length (SRL) of the species. Blue color indicates forbs and green color indicates grasses. Data represent mean ± SE. Initial WSA were 29% in disintegrated and 44% in aggregated soil. The relationship between SRL and %WSA is significant in aggregated soil (solid line, *r*² = .360, *p *= .003) but not in disintegrated soil (dashed line, *r*² = .000, *p *= .962). The interaction between the effects “soil structure” and “SRL” significantly affects the formation of WSA (*p *= .037) in a linear mixed‐effects model with the random effect “plant species” (see Table 1)

In the training phase when using SRL as the predictive trait, we found no effect of soil structure on plant biomass production (Table 1), but there was a difference in biomass allocation. Plants allocated significantly more biomass aboveground with increasing SRL on disintegrated soil than on aggregated soil (Fig. 3). When using PFT as the predictor, the soil structure also as a single factor significantly affected the belowground biomass production and thereby the biomass allocation (Table S4) with grasses allocating more biomass aboveground overall. The formation of WSA was significantly affected by the PFT (Table S4) with forbs producing a higher percentage of WSA than grasses (Fig. 4). We found a negative correlation between SRL and WSA formation on aggregated soil that significantly differed from disintegrated soil (Fig. 4). The initial soil structure of the disintegrated soil with 29% WSA did not change overall but included some plants (like *D. glomerata*) which actually decreased soil aggregation down to <20% WSA, while others (like *L. vulgare*) increased it to 33% WSA. The aggregated soil with initially 44% WSA increased in aggregation with an overall mean of 48%. We could rarely detect decreases in WSA in the aggregated soil (the strongest was 2% by *B. media*) while plants with low SRL as *D. carota* increased aggregation up to 55% WSA (Fig. 4). The AM colonization as well as the number of arbuscules differed significantly between the soil structure level and was higher on the disintegrated soil (mean AM colonization in aggregated soil: 37% <48% in disintegrated soil, Tables S2 and S3, Fig. S1) indicating that the disintegrating process did not negatively influence mycorrhizal fungal root colonization. Total colonization as well as number of arbuscules and vesicles was significantly negatively correlated with SRL (Table S2), and forbs were highly significantly more colonized than grasses (Table S3, Fig. S1).

**Table 1:** Summary of the linear mixed‐effects models for the training (A) and feedback (B) phases

A											
Effect	*df*	b		ab		bb		Allocation		WSA	
		*F*	*p*	*F*	*p*	*F*	*p*	*F*	*p*	*F*	*p*
Soil	1	0.016	.900	0.168	.682	0.322	.571	0.104	.747	102.871	**<.001**
SRL	1	0.044	.839	0.121	.735	0.472	.508	1.422	.261	2.331	.158
Soil × SRL	1	0.749	.388	1.273	.261	5.267	**.023**	8.508	**.004**	4.423	**.037**

B									
Effect	*df*	b		ab		bb		Allocation	
		*F*	*p*	*F*	*p*	*F*	*p*	*F*	*p*
History	1	8.602	**.004**	14.388	**<.001**	0.850	.357	0.504	.478
Soil	1	0.470	.493	0.005	.944	2.176	.142	0.105	.746
History × soil	1	0.018	.894	0.026	.872	0.231	.631	2.225	.137
Home									
Soil	1	4.262	**.041**	0.792	.375	9.746	**.002**	11.205	**.001**
SRL	1	0.311	.591	2.899	.123	1.502	.215	19.316	**.002**
Soil × SRL	1	4.453	**.036**	1.384	.241	8.064	**.005**	8.596	**.004**
Away									
Soil	1	0.103	.748	0.002	.968	0.453	.502	0.029	.864
SRL	1	0.194	.670	2.489	.149	2.332	.161	19.950	**.002**
Soil × SRL	1	0.011	.918	0.009	.926	0.103	.749	0.457	.450

Main effect of history (home vs. away) as well as main and interactive effects of soil structure (aggregated vs. disintegrated) and specific root length (SRL) on dry biomass (b, total biomass; ab, aboveground biomass; bb, belowground biomass; Allocation, log(ab/bb) and water‐stable aggregates (WSA) are estimated. SRL was fitted as a continuous variable that is constant per plant species. Degrees of freedom (df), *F* values and *p* values from ANOVA are presented. Significant values (*p* < .05) are presented in bold.

### 3.2 Feedback phase

In the feedback phase, we found a significant effect of soil history (home vs. away) on total plant biomass as well as on aboveground biomass (Table 1) with plants experiencing a negative conspecific feedback overall. Belowground biomass was not significantly affected by the soil history, so all changes in total biomass took primarily place in the aboveground biomass. The effects of heterospecific‐trained soil microbes on biomass production did not differ between disintegrated and aggregated soil (Table 1, Table S4). The only effect pronounced within the heterospecific treatment was a correlation of biomass allocation and SRL (Table 1), as well as a significant effect of PFT on biomass allocation (Table S4) which also occurred in conspecific‐trained soil. Biomass allocation in the heterospecific treatment was positively correlated with SRL (*r* = .588, *p *< .001) indicating that plants with higher SRL allocated more biomass aboveground irrespective of training soil structure being aggregated or disintegrated.

**Figure 5:**
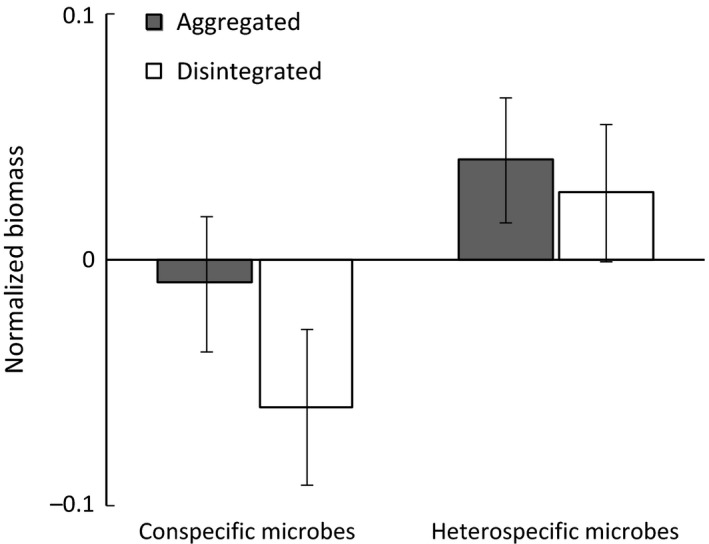
Feedback phase. Effect of different training soil structure on plant biomass after inoculation with conspecific or heterospecific microbes, respectively. For a better visualization, biomass data are normalized per plant by (x‐species mean)/species mean over the entire experiment to account for species‐specific differences. Data represent mean ± SE. For conspecific microbes, the soil structure significantly affects plant biomass production (*p *= .041) in a linear mixed‐effects model with the additional fixed effect “SRL” and the random effect “plant species” (see Table 1)

**Figure 6:**
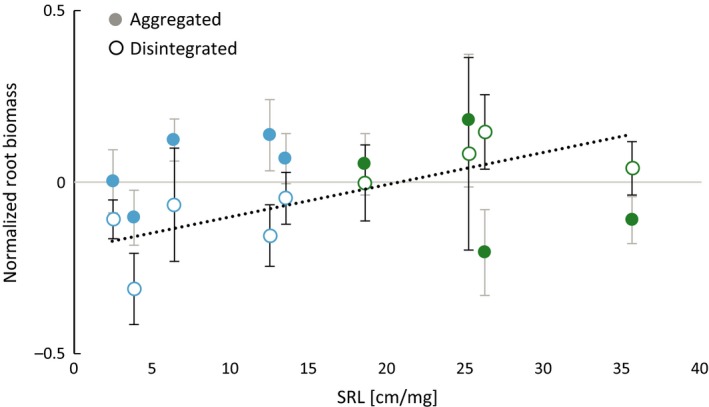
Feedback phase. Effect of conspecific soil microbes. Displayed is the specific root length (SRL) as a mediating factor of root biomass production. For a better visualization, biomass data are normalized per plant by (x‐species mean)/species mean over the entire experiment to account for species‐specific differences. Blue color indicates forbs and green color indicates grasses. Data represent mean ± SE. The relationship between SRL and normalized dry root biomass is significant in disintegrated soil (dashed line, *r*² = .603, *p *= .025) but not in aggregated soil (*r*² = .068, *p *= .239). The interaction between the effects “soil structure” and “SRL” significantly affects the root biomass production (*p *= .005) in a linear mixed‐effects model with the random effect “plant species” (see Table 1)

The biomass effects of conspecific‐trained soil microbes—being negative overall—were significantly affected by the soil structure of the training phase in the model with SRL as the predictive trait (Table 1). With the predictive factor PFT, the single effect of soil was not significant (Table S4). Under the influence of conspecific microbes from disintegrated soil, plants produced less biomass than with those from aggregated soil (i.e., negative conspecific feedback was stronger; Fig. 5). The results of the linear mixed‐effects models illustrated a significant interaction between the former soil structure and the SRL (Table 1) and the PFT, respectively (Table S4), in affecting biomass. With conspecific microbes from disintegrated soil, biomass production was mediated by SRL (*r* = .230, *p *= .039). This effect was mainly caused by differences in root biomass production, which therefore also resulted in significant effects on biomass allocation (Table 1, Table S4). In disintegrated soil, plants with higher SRL produced relatively more root biomass (Fig. 6) compared to all other treatments resulting also in a less negative feedback. Plants with a SRL up to app. 20 cm/mg experienced negative effects on root biomass while species with a SRL higher than that benefitted from conspecific microbes from disintegrated soil in comparison with all other treatments. This effect was most prominent in belowground biomass (Fig. 6) but was also reflected in total biomass production. The SRL clearly separated forbs (low SRL) and grasses (high SRL) (Table S5). The mediating effect of SRL could only be observed with conspecific microbes from disintegrated soil. Biomass production under the influence of conspecific microbes from aggregated soil was not correlated with SRL.

Summarizing these observations, the aboveground biomass showed feedback effects irrespective of soil structure while within the conspecific feedback, the soil structure affected belowground and thereby total biomass production mediated by SRL which discriminates between forbs and grasses.

## 4 Discussion

As far as we know, soil structure has never been taken explicitly into account when investigating PSF. The major conclusion we can draw from our experiment is that soil structure can have a profound influence on the outcome of PSF. We initially hypothesized a stronger negative feedback on disintegrated soil due to a higher host specificity and virulence of pathogens under these conditions as well as to lower dependency on mutualists. Our results support this hypothesis. The SRL was not measured within the experiment because reciprocal effects would have made it impossible to distinguish cause and effect. It can be criticized that with such an approach, the SRL is not directly linked to the effects observed as it only represents a potential and not an actual morphological character of the experimental plants. This is a general issue with trait‐based approaches using data from databases (Cordlandwehr et al., 2013) that we tried to diminish to some extent using data of an independent experiment with the same seed material representing a species‐specific trait not effected by any of our treatments. The correlation of SRL, as a suggested explanatory variable for the observed effects, turned out to be a significant mediator of the training process in disintegrated soil. The SRL separates the two PFTs (forbs and grasses) which on their own explain most of the effects observed. It is a challenge to identify the mechanisms underlying a process that coincides with clear PFTs. Furthermore, if a mechanistic explanation has been found, it is most likely not the only factor that drives the process. It is still obvious that a significant correlation of SRL with various effects during training and feedback phase indicates a direct mechanistic relation and explains an important component of the different effects observed for grasses and forbs. To discuss the impact of SRL on PSF in different soil structure, the two phases of the experiment need to be examined in detail.

### 4.1 Training phase

The results of the training phase illustrate the physical effects of soil structure on plant performance. The soil and its microbial community was initially the same except for the impact of the disintegrating process. Therefore, the effects observed were caused by physical structure of the soil (either directly on plants or indirectly via different microbial performance during the training phase due to soil structure) or by different nutrient availability due to aggregation status. Plant‐available phosphorus as well as nitrogen were slightly elevated in the disintegrated soil, but as plant biomass was not affected by soil structure, this seems to not have influenced plant performance directly. Therefore, we assume that the effects observed are not primarily caused by nutrient availability but by physical structure of the soil. The high colonization by AM fungi in both treatments showed that the process of disintegrating did not destroy the large mycorrhizal fungal propagules and so most likely did also no harm to propagules of other microbiota of comparable or smaller size. Plants with higher SRL (grasses) showed less AM colonization, and all plants were more colonized in disintegrated soil with even more arbuscules. Rillig and Steinberg (2002) found that AM colonization did not differ when extraradical hyphae colonized substrate of different aggregate sizes (simulated by glass beads) with roots growing in vitro without access to the hyphal compartment. If differences in AM colonization were to arise from differences in soil nutrients, we would expect a stronger colonization in the aggregated soil with less available nutrients, which was not the case. Furthermore, it is known that a simultaneous increase in P and N buffers the effects a single P increase would induce for arbuscular mycorrhizal colonization of plants (Camenzind et al., 2014; Johnson, 2010). We therefore suggest that the difference in colonization in our experiment is an indirect effect of the soil structure due to root performance: as roots can grow more easily in soil with larger aggregates and resulting larger pores, this could lead to a lower dependency on mycorrhizal partners especially for water uptake. The gradient of SRL clearly separated forbs and grasses in our experiment with the latter having the higher SRL, which is also known from the literature (Craine, Froehle, Tilman, Wedin, & Chapin, 2001). Grasses, at least members of the Pooideae subfamily (C3), are known to have a low mycorrhizal responsiveness (Reinhart et al., 2012). This supports our result of lower colonization rates in plants with higher SRL irrespective of the soil.

In the training phase, total plant biomass was not affected by soil structure. But on disintegrated soil, plants with a higher SRL and accordingly grasses allocated proportionally more biomass aboveground. The general linear mixed‐effects model containing the factor “PFT” reveals a single effect of soil structure with a general biomass allocation toward aboveground plant parts on disintegrated soil. Actually, Figure 3 clearly shows that the effect of soil structure on biomass allocation cannot be observed in most forbs with low SRL and is therefore mainly driven by the interaction with SRL or PFT, respectively. Following the concept of a “functional equilibrium” of biomass allocation (Brouwer, 1963; Poorter et al., 2012), this could be translated to differences in belowground limitation along a SRL gradient on disintegrated soil. We cannot exclude whether that limitation is by soil nutrients or not, but the fact that AM colonization was higher in disintegrated soil, which contained slightly more plant‐available phosphorus does not point in this direction. The fact that especially grasses (with higher SRL and less AM colonization) produced less root biomass on disintegrated soil rather raises the question whether there could be different phylogenetically conserved strategies in coping with soil structure or whether there are other mechanisms causing this effect. Studies on the effect of soil structure on root performance are scarce, but results obtained using maize seedlings suggest that plants can alter allocation patterns toward roots in response to larger soil aggregates, which was unrelated to nutrient status of the plants but appears to be a direct response (Alexander & Miller, 1991). We propose that the aggregated soil is easily penetrable by roots irrespective of the SRL (in the range of our experimental plants, see Table S5) with nutrients to some degree being sequestered inside aggregates forcing the plants to allocate biomass belowground for soil exploration and nutrient acquisition. In the disintegrated soil, the SRL (mainly caused by root fineness) determines the ability of exploring the soil structure with smaller pores where nutrients are more equally distributed and less sequestered inside large aggregates (Linquist, Singleton, Yost, & Cassman, 1997). That causes an advantage for plants with higher SRL to obtain soil nutrients and reduces the need for an additional investment in root biomass.

We know that apart from abiotic factors such as soil texture (Wick, Huzurbazar, & Stahl, 2009), plant roots and microbiota such as mycorrhizal fungi can have a profound influence on levels of soil aggregation (Rillig & Mummey, 2006; Six et al., 2004). In experimental plant communities of the Jena experiment, grasses (with high SRL) had the strongest positive contribution to soil aggregation, as compared to forbs and legumes (Pérès et al., 2013). We found the opposite when testing the effect of SRL on the formation of WSA. In the aggregated soil, the formation of WSA was negatively correlated with SRL. This could be either due to root penetration resulting in destruction of macroaggregates (Angers & Caron, 1998; Materechera, Kirby, Alston, & Dexter, 1994; Six et al., 2004) or to reduced length of AM fungal hyphae. As AM hyphae can increase soil aggregation in various ways (Rillig & Mummey, 2006; Tisdall & Oades, 1982), the lower colonization rate of plants with higher SRL is very likely to negatively affect the formation of WSA. In the disintegrated soil, the effects of the training phase varied from slight elevation to clear decrease in aggregation. The process of disintegration led to the destruction of macroaggregates (250 μm – 4 mm) falling apart into stable microaggregates (<250 μm). Angers, Recous, and Aita (1997) showed that the formation of highly water‐stable microaggregates takes place within macroaggregates. Six, Elliott, Paustian, and Doran (1998), Six et al. (2004) concluded that disturbance (such as tillage or other processes unfolding disintegrating forces) reduces the amount of macroaggregates resulting in a reduced formation of new microaggregates. This could be a possible explanation for the lacking net formation of new aggregates in our disintegrated soil. We cannot identify the mechanism behind the further disintegration caused by some plants, but we suppose that the partially destroyed aggregates were damaged to a point where they were predestined to further fall apart to some extent. This effect was not correlated to SRL, so there seems to be other plant‐ or soil microbial community‐specific traits driving the process of further disintegration.

### 4.2 Feedback phase

At the beginning of the feedback phase, the soil aggregation was equal in all treatments. Therefore, the results observed were caused by the biological components of the trained soil primarily reflecting soil microbial differences due to initial soil structure level of the training phase.

Plants growing with heterospecific microbes showed no response to the microbes originating from different soil structure levels of the training phase. The SRL was positively correlated with the biomass allocation toward aboveground parts of the plant. This effect is comparable to the one we found in the training phase on disintegrated soil where microbes from field soil represent a predominantly heterospecific‐trained inoculum. The autoclaving of the uniform soil of the feedback phase as well as the mixing with sand is likely to have disintegrated the soil structure favouring grasses with their high SRL and allowing them to allocate more biomass aboveground like we argued above to account for this effect in the training phase.

Plants growing with conspecific microbes produced less biomass than the ones in the away treatment. The conspecific feedback was therefore negative, leading to the conclusion that host‐specific antagonists likely accumulated during the training phase in both soil structure levels. Following the basic concept of PSF, this translates to an expected stabilization of plant diversity by coexistence (Bever, 2003) in a community irrespective of soil structure. However, the negative conspecific feedback was most pronounced with microbes from disintegrated soil. The fact that this effect was only significant in the general linear mixed‐effects model with SRL strengthens the argument that the SRL was a significant mediator of this effect with plants with a low SRL experiencing the most negative feedback. Following our initial hypothesis, this could be either due to more negative effects by pathogens or less positive effects by mutualists. As plants with lower SRL had a high percentage of colonized roots by AM fungi on disintegrated soil in the training phase, a reduced positive influence of mutualists in the respective soils of the feedback phase is not likely. It seems more plausible that host‐specific pathogens were the drivers of the reduced biomass production observed in the feedback phase. As PSF is a complex reciprocal process, it is hard to distinguish whether the fungal or the plant partner (or any other biological participant) is responsible for an observed effect. Taking into account that during training on disintegrated soil, belowground biomass production and biomass allocation were correlated with SRL, it seems most likely that the effects we observed were caused by different plant performance during training along a SRL gradient. We argue that the physical structure of disintegrated soil (with smaller pores and more equally distributed nutrients) represented a disadvantage for plants with low SRL—namely forbs—in terms of effective soil exploration. This may have caused the need to allocate relatively more biomass belowground and increase exposure to soil pathogens. The consequence is a conspecific pathogen enrichment causing negative biomass effects in the feedback phase. Our initially hypothesized concept that specialist pathogens might perform better in disintegrated soil seems to be part of a much more complex interaction.

Aboveground biomass in the feedback phase was not affected by the former soil structure nor by the SRL. All changes related to soil structure took place belowground reflected by significant effects on biomass allocation in the conspecific inoculated plants. The stronger negative conspecific feedback effect with microbes from disintegrated soil (Fig. 5) therefore mainly arose from a reduction of belowground biomass especially in plants with low SRL. A plausible explanation for this would be that plants have lost roots to species‐specific pathogens and that this was a higher burden on plants with relatively expensive, low‐SRL roots: these plants may have invested more in AM symbiosis than in root biomass (Veresoglou, Menexes, & Rillig, 2012), which may make these roots even more expensive to replace. On the contrary, plants with higher SRL may compensate root loss to pathogens more easily by producing new fine roots. Mommer et al. (2011) suggested that fine roots are relatively cheap in terms of biomass investment to explain the observed advantage for plants with high SRL (experiment on *A. odoratum*) in nutrient foraging. Our results appear to support de Kroon et al. (2012) who suggested that the responses of roots to soil microbes are underestimated regarding their impact on plant community dynamics. More specifically, we show that feedbacks to soil structure are primarily reflected in belowground parts of plants, while feedbacks to conspecific versus heterospecific training are mainly reflected in aboveground plant parts.

## 5 Conclusion

This study has been the first to examine the effects of soil structure on PSF. We find the complex interaction between plant roots and microbial colonizers—being pathogenic or mutualistic—that creates PSF is to some extent dependent on soil structure, leading to stronger negative feedback in disintegrated soil. Furthermore, our results indicate that this effect is strongly mediated by a plant‐specific root morphological trait, that is, SRL that coincides with the distinction between grasses and forbs. It has previously been argued that plant traits could explain PSF effects in grassland communities (Baxendale et al., 2014). However, our finding of more negative feedback for low‐SRL plants in disintegrated soil goes against the expectation that finer roots are more susceptible to fungal pathogens (Bever, 1994; Newsham et al., 1995). A potential explanation is that higher nutrient scavenging capabilities and replaceability of high‐SRL roots in the small‐pored disintegrated soil more than counteracts this expected stronger negative feedback. Clearly, more research is needed on the relationship between SRL and PSF, as well as on the degree to which this mechanistic trait contributes to the differences between grasses and forbs, and soil structure is a primary candidate of potential moderator variables to take into account. It has been shown that soil structure in terms of WSA and size distribution is positively correlated with succession (Cheng, Xiang, Xue, An, & Darboux, 2015; Duchicela, Sullivan, Bontti, & Bever, 2013; Erktan et al., 2015). In that context, our findings raise the question whether plants with lower SRL may be more limited by PSF in early successional stages. This question has not been addressed explicitly so far, but Erktan et al. (2015) found SRL to be negatively correlated with aggregate stability along a successional gradient. Zangaro et al. (2008), Zangaro, Alves, Lescano, Ansanelo, and Nogueira (2012), Zangaro et al. (2014) found high SRL and low root diameter to be associated with early successional stages in different ecosystems in Brazil. Regarding our results, it would be an exciting research avenue to study how root traits affect soil structure, PSF, and the potential interaction between these factors in a vegetational succession context.

## Data Accessibility

Data on SRL, soil analysis, and root colonization: uploaded as online supporting information. Plant biomass data: to be submitted to dryad.

## Conflict of Interest

None declared.
