# Supplementary material for: The interplay between soil structure, roots, and microbiota as a determinant of plant–soil feedback
Source: Ecol Evol. 2016 Oct 5;6(21):7633–44. doi: 10.1002/ece3.2456 (PMC6093149; doi:10.1002/ece3.2456)
Supplement: Supplementary file 1 [file ECE3-6-7633-s001.docx]

**Supporting Information**

**Table S1.** Nutrient analysis of the soil used in the training phase. Data represent mean ± SE.

| soil | pH (20°C) | Pdl [mg/100g] | Pcal [mg/100g] | NO3-N [mg/100g] | NH4-N [mg/100g] |
| --- | --- | --- | --- | --- | --- |
| aggregated | 6.92 ± 0.04 | 4.68 ± 0.02 | 2.96 ± 0.08 | 3.91 ± 0.03 | 0.01 ± 0.00 |
| disintegrated | 6.97 ± 0.03 | 5.25 ± 0.09 | 3.50 ± 0.05 | 5.20 ± 0.06 | 0.03 ± 0.00 |

**Table S2.** Summary of the linear mixed effects model (lmer) testing for main and interactive effects of soil structure and specific root length (SRL) on hyphal colonization, arbuscules and vesicles during the training phase. SRL was fitted as a numeric effect. Degrees of freedom (df), *F* values and *p* values from ANOVA are presented. Significant values (p < 0.05) are presented in bold. Downward arrows indicate a negative correlation.

|  |  | colonization | | arbuscules | | vesicles | |
| --- | --- | --- | --- | --- | --- | --- | --- |
| effect | d.f. | *F* | *p* | *F* | *p* | *F* | *p* |
| soil | 1 | 11.243 | **0.001** | 5.769 | **0.018** | 3.237 | 0.075 |
| SRL | 1 | 27.410 | **<0.001↓** | 30.016 | **<0.001↓** | 25.868 | **<0.001↓** |
| soil*SRL | 1 | 1.312 | 0.255 | 1.108 | 0.295 | 1.012 | 0.317 |

**Table S3** Summary of the linear mixed effects model (lmer) testing for main and interactive effects of soil structure and plant functional type (PFT) on hyphal colonization, arbuscules and vesicles during the training phase. Degrees of freedom (df), *F* values and *p* values from ANOVA are presented. Significant values (*p* < 0.05) are presented in bold.

|  |  | colonization | | arbuscules | | vesicles | |
| --- | --- | --- | --- | --- | --- | --- | --- |
| effect | d.f. | *F* | *p* | *F* | *p* | *F* | *p* |
| soil | 1 | 16.833 | **<0.001** | 7.487 | **0.007** | 1.780 | 00.185 |
| PFT | 1 | 61.492 | **<0.001** | 115.671 | **<0.001** | 30.684 | **<0.001** |
| soil*PFT | 1 | 0.757 | 0.386 | 0.012 | 0.914 | 2.881 | 0.093 |

**Table S4.** Summary of the linear mixed effects models for the training (A) and feedback (B) phase using the plant functional type ("PFT"; grasses vs. forbs) as explanatory factor. Main effect of history (home vs. away) as well as main and interactive effects of soil structure (aggregated vs. disintegrated) and PFT on dry biomass (b, total biomass; ab, aboveground biomass; bb, belowground biomass; ln(a/bb), biomass allocation) and water stable aggregates (WSA) are estimated. Degrees of freedom (df), *F* values and *p* values from ANOVA are presented. Significant values (p < 0.05) are presented in bold.

| **A** |  |  | |  |  | |  |  | |  |  | |  |  | |
| --- | --- | --- | --- | --- | --- | --- | --- | --- | --- | --- | --- | --- | --- | --- | --- |
|  |  | b | |  | ab | |  | bb | |  | ln(a/bb) | |  | WSA | |
| effect | d.f. | *F* | *p* |  | *F* | *p* |  | *F* | *p* |  | *F* | *p* |  | *F* | *p* |
| soil | 1 | 2.031 | 0.156 |  | 1.299 | 0.256 |  | 9.724 | **0.002** |  | 22.337 | **<0.001** |  | 218.702 | **<0.001** |
| PFT | 1 | 0.153 | 0.704 |  | 0.521 | 0.487 |  | 2.085 | 0.179 |  | 4.717 | 0.055 |  | 5.033 | **0.049** |
| soil*PFT | 1 | 2.424 | 0.122 |  | 0.605 | 0.438 |  | 8.734 | **0.004** |  | 14.295 | **<0.001** |  | 1.480 | 0.226 |

| **B** |  |  | |  |  | |  |  | |  |  | |
| --- | --- | --- | --- | --- | --- | --- | --- | --- | --- | --- | --- | --- |
|  |  | b | |  | ab | |  | bb | |  | ln(a/bb) | |
| effect | d.f. | *F* | *p* |  | *F* | *p* |  | *F* | *p* |  | *F* | *p* |
| home | | | | | | | | | | | | |
| soil | 1 | 0.099 | 0.753 |  | 0.088 | 0.767 |  | 1.092 | 0.298 |  | 1.691 | 0.195 |
| PFT | 1 | 0.250 | 0.629 |  | 2.038 | 0.187 |  | 1.007 | 0.342 |  | 12.499 | **0.006** |
| soil*PFT | 1 | 4.563 | **0.034** |  | 1.507 | 0.221 |  | 7.978 | **0.005** |  | 8.633 | **0.004** |
| away | | | | | | | | | | | | |
| soil | 1 | 0.199 | 0.657 |  | 0.012 | 0.912 |  | 0.512 | 0.476 |  | 0.660 | 0.418 |
| PFT | 1 | 0.112 | 0.746 |  | 1.526 | 0.248 |  | 1.607 | 0.237 |  | 10.098 | **0.011** |
| soil*PFT | 1 | 0.042 | 0.838 |  | 0.154 | 0.696 |  | 0.014 | 0.905 |  | 0.958 | 0.329 |

**Table S5.** Mean specific root length (SRL) of the 10 species used in the experiment. These data originate from a separate experiment with seeds from the same field collection than in the presented feedbackexperiment.

| \| Species \| SRL [cm/mg] \| \| \| --- \| --- \| --- \| \| *Plantago major* \| 2.48 \| **forbs** \| \| *Daucus carota* \| 3.81 \| \| *Centaurea jacea* \| 6.33 \| \| *Leucanthemum vulgare* \| 12.46 \| \| *Plantago lanceolata*  *Taraxacum officinale* \| 13.44  14.95 \| \| *Dactylis glomerata* \| 18.49 \| **grasses** \| \| *Briza media* \| 25.14 \| \| *Anthoxantum odoratum* \| 26.16 \| \| *Holcus lanatus* \| 35.55 \| |  |
| --- | --- | --- | --- | --- | --- | --- | --- | --- | --- | --- | --- | --- | --- | --- | --- | --- | --- | --- | --- | --- | --- | --- | --- | --- |

**
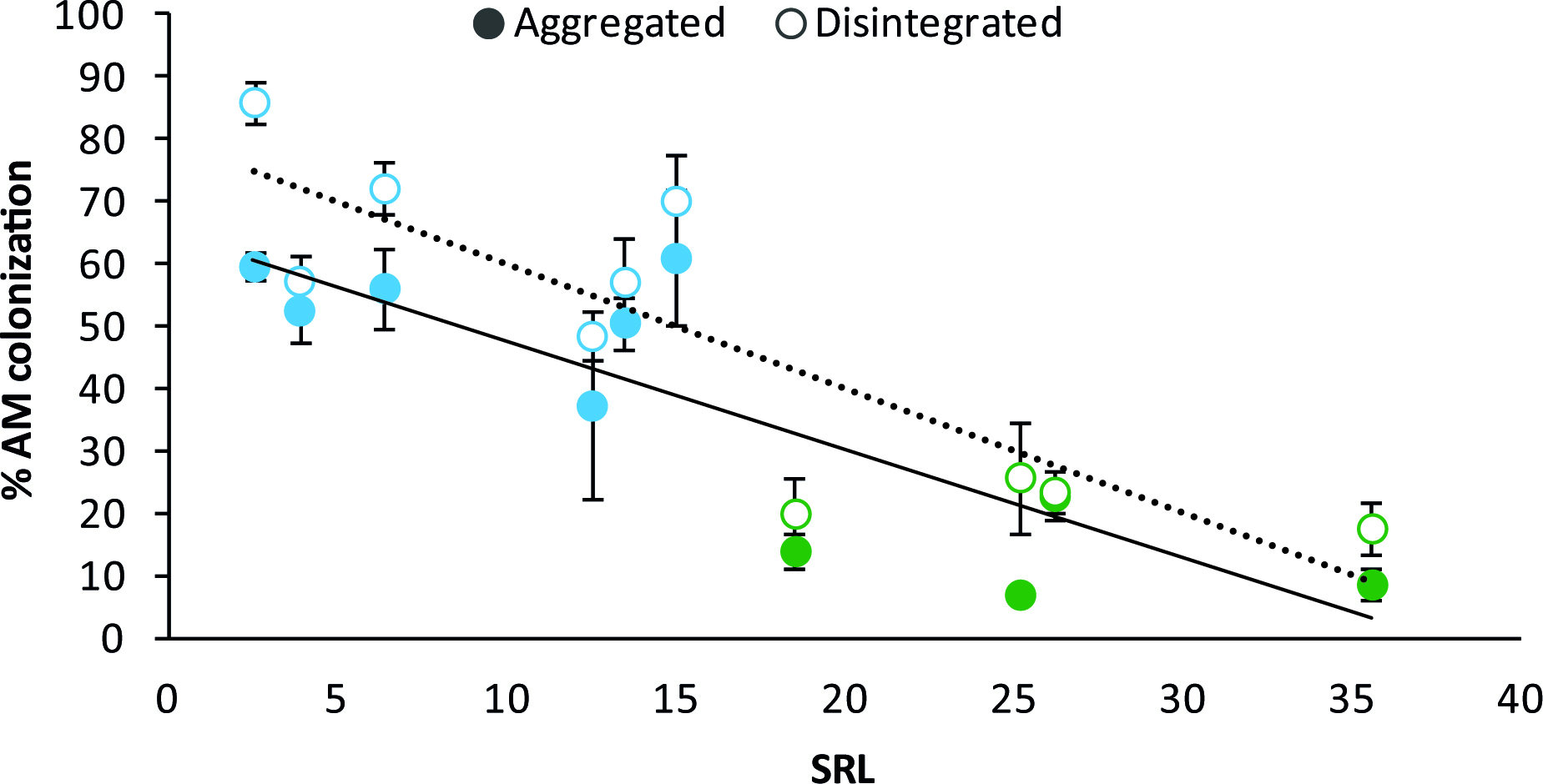
**

**Fig. S1.** Training phase. AM-colonization of plant roots in the two soil structure levels in correlation to the specific root length (SRL) of the species. Blue colouring indicates forbs, green colouring indicates grasses. Data represent mean ± SE. The relationship of SRL and % AM colonization is significant in aggregated soil (solid line, *r*²=0.713, *p<*0.001) as well as in disintegrated soil (dashed line, *r*²=0.732, *p*<0.001).
